# mTORC1 activation decreases autophagy in aging and idiopathic pulmonary fibrosis and contributes to apoptosis resistance in IPF fibroblasts

**DOI:** 10.1111/acel.12514

**Published:** 2016-08-26

**Authors:** Yair Romero, Marta Bueno, Remedios Ramirez, Diana Álvarez, John C. Sembrat, Elena A. Goncharova, Mauricio Rojas, Moisés Selman, Ana L. Mora, Annie Pardo

**Affiliations:** ^1^ Facultad de Ciencias Universidad Nacional Autónoma de México Av Universidad 3000 México DF CP 04510 México; ^2^ Division of Pulmonary Allergy and Critical Care Medicine University of Pittsburgh 200 Lothrop St Pittsburgh PA 15261 USA; ^3^ Vascular Medicine Institute Pulmonary Division University of Pittsburgh 200 Lothrop St Pittsburgh PA 15261 USA; ^4^ Instituto Nacional de Enfermedades Respiratorias Ismael Cosío Villegas México Tlalpan 4502 DF CP 14080 México

**Keywords:** aging, apoptosis, autophagy, idiopathic pulmonary fibrosis, lung fibroblast, mTOR pathway

## Abstract

Idiopathic pulmonary fibrosis (IPF) is a chronic, progressive, and usually lethal disease associated with aging. However, the molecular mechanisms of the aging process that contribute to the pathogenesis of IPF have not been elucidated. IPF is characterized by abundant foci of highly active fibroblasts and myofibroblasts resistant to apoptosis. Remarkably, the role of aging in the autophagy activity of lung fibroblasts and its relationship with apoptosis, as adaptive responses, has not been evaluated previously in this disease. In the present study, we analyzed the dynamics of autophagy in primary lung fibroblasts from IPF compared to young and age‐matched normal lung fibroblasts. Our results showed that aging contributes for a lower induction of autophagy on basal conditions and under starvation which is mediated by mTOR pathway activation. Treatment with rapamycin and PP242, that target the PI3K/AKT/mTOR signaling pathway, modified starvation‐induced autophagy and apoptosis in IPF fibroblasts. Interestingly, we found a persistent activation of this pathway under starvation that contributes to the apoptosis resistance in IPF fibroblasts. These findings indicate that aging affects adaptive responses to stress decreasing autophagy through activation of mTORC1 in lung fibroblasts. The activation of this pathway also contributes to the resistance to cell death in IPF lung fibroblasts.

## Introduction

Idiopathic pulmonary fibrosis (IPF) is a highly lethal lung disease of unknown etiology characterized by activation of alveolar epithelial cells, fibroblast/myofibroblast proliferation, and activation with exacerbated deposit of extracellular matrix (ECM) resulting in the gradual destruction of the lung architecture (Selman *et al*., 2001; King *et al*., 2011). In this sequence of pathological events, fibroblasts/myofibroblasts are usually organized in a distinctive foci and it has been suggested to be resistant to apoptosis (Kazufumi *et al*., 1997).

IPF is a multifactorial disease that likely results from complex interactions between genetic and environmental factors (Taskar & Coultas, 2006) (Fingerlin *et al*., 2013). The most important environmental risk factors are cigarette smoking and exposure to metal and wood dust, while several gene variants associated with host defense, cell–cell adhesion, and DNA repair contribute to IPF risk. Importantly, IPF occurs in middle‐aged and elderly adults and the incidence and prevalence increase markedly with each decade of life; actually, two‐thirds of patients with sporadic IPF are older than 60 years at the time of presentation with a mean age of 66 years at the time of diagnosis (King *et al*., 2011) (Raghu *et al*., 2006). Likewise, patients with familial IPF display autosomal dominant inheritance with age‐dependent penetrance, and abnormally short telomeres have been observed in both familial IPF with telomerase mutations and sporadic IPF (Armanios & Blackburn, 2012). Importantly, telomerase deficiency and telomere attrition trigger telomere dysfunction‐mediated alveolar stem cell replicative senescence, thereby driving pulmonary premature aging, reducing the regenerative capacity, and increasing myofibroblast activity and fibrosis (Chen *et al*., 2015). Furthermore, senescence of alveolar epithelial cells and fibroblasts has been found in this disease strengthening the mechanistic links between telomere shortening, cell senescence, and IPF (Minagawa *et al*., 2011; Hecker *et al*., 2014).

Although age is a determinant factor of the pathogenesis of IPF, the molecular mechanisms of the aging process that influence the susceptibility to develop this disease and its clinical progression are still uncertain (Selman *et al*., 2010; Selman & Pardo, 2014).

Aging is characterized by increasing the risk of disease and death. In this process, there is a progressive reduction of biological functions and less resistance to multiple stressors. In this context, a pivotal anti‐aging pathway is autophagy (Lopez‐Otin *et al*., 2013). Macroautophagy (referred as autophagy) is a highly conserved process that participates in maintaining the energy resources and quality control by degradation of unnecessary elements. As an adaptive response, autophagy is able to relief stressful conditions as starvation, hypoxia, endoplasmic reticulum stress, oxidative stress, etc. (He & Klionsky, 2009). Among the sensors that regulate autophagy, the serine/threonine protein kinase mammalian target of rapamycin (mTOR) plays a central role in promoting growth in the presence of nutrients and maintaining low levels of autophagy. But, when cells are under starvation condition, mTORC1 is switched off and autophagy is activated (Laplante & Sabatini, 2012). Importantly, it has been recently shown that mTOR pathway drives the senescence‐associated secretory phenotype which is suppressed by rapamycin (Laberge *et al*., 2015).

There are few reports that evaluate the relationship between autophagy and IPF and most of them agree that autophagy activity is reduced in IPF lungs, suggesting that this decrease may contribute to the activation of pro‐fibrotic responses (Patel *et al*., 2012; Araya *et al*., 2013; Ricci *et al*., 2013; Nho & Hergert, 2014). However, these studies lack the corresponding age‐matched controls and shelve the dynamic connection between autophagy and apoptosis in the stress responses. In the present study, we explored the effect of aging in autophagy activity and its implication in the pathogenesis of IPF.

## Results

### Aging decreases autophagy activity in fibroblasts from patients with IPF

To evaluate the effect of aging on autophagy, normal human lung fibroblasts were sorted out among ‘young’ normal donors [mean±SD 29 ± 11 years (*n* = 6)] and ‘old’ normal donors [67 ± 7 years (*n* = 6)]. IPF fibroblasts were obtained from 6 patients with a mean age of 66 ± 4 years (Table S1).

**Figure 1:**
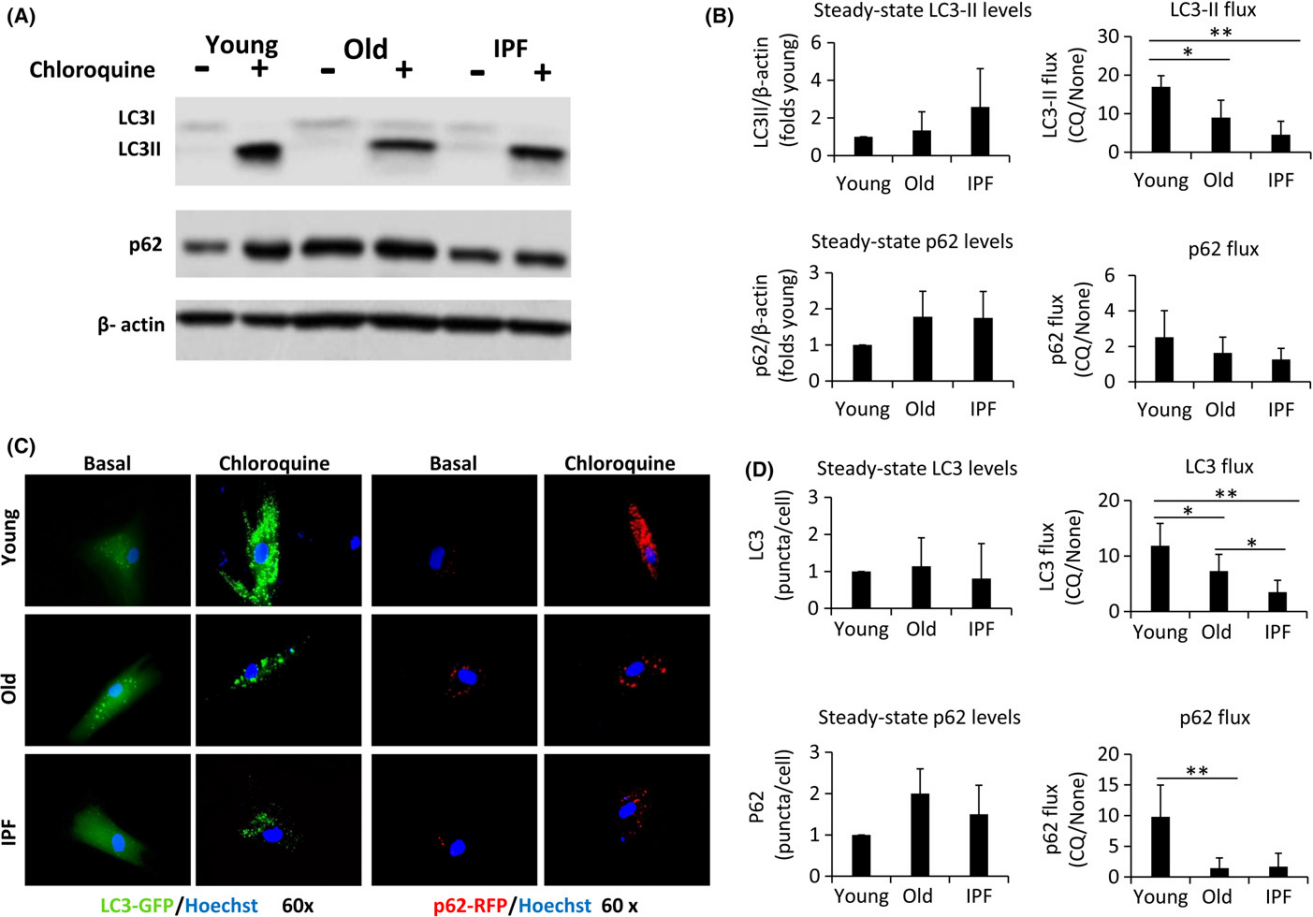
Aging decreases autophagy flux in IPF fibroblasts. (A) Western blots of LC3 and p62 after 24 h with/without chloroquine treatment (20 μM) in human fibroblasts derived from young, old, and IPF lungs. β‐actin was used as a loading control. (B) Densitometric analysis representing LC3‐II and p62 steady‐state and flux levels. Each bar represents the mean ± SD of 3 different lines of lung fibroblasts. (C) Fluorescence microscopic images show LC3 and p62 distribution. Baculovirus infection of LC3‐GFP and p62‐RFP in human fibroblasts from young, old, and IPF lungs under basal conditions and after 24 h with chloroquine treatment (20uM). Hoechst was added to nuclei stain. LC3 and p62 puncta were quantified in D; each bar represents the mean ± SD of 3 different cell lines for each group (n > 30). *P < 0.05, **P < 0.01 two‐tailed Student's *t*‐test.

Autophagy flux involves a dynamic autophagosome formation and degradation that can be estimated by monitoring the protein levels of LC3 in the presence and absence of lysosomal inhibitors (Tanida *et al*., 2005). LC3 has two forms in the cell: LC3‐I in the cytosol and LC3‐II which is associated specifically with the autophagosome membrane. We cultured the fibroblasts from the three groups in the presence of the lysosomal inhibitor, chloroquine (CQ), to determine the level of autophagy through the amount of LC3‐II. Figure**** 1A exemplifies a representative Western blot of one young, one old, and one IPF cell lines. Figure**** 1B represents the densitometric analysis of three different cell lines for each condition showing the steady‐state LC3‐II levels and the LC3‐II flux. A significantly higher LC3‐II flux was observed in normal young human lung fibroblasts compared to normal old and IPF fibroblasts after 24 h of CQ treatment (*P* < 0.05).

Another marker of autophagy is p62, an autophagic cargo involved in the recognition of aggregated proteins. No significant differences were observed in the p62 steady‐state levels or in p62 flux (Fig. 1A,B).

Autophagy was further examined using GFP‐LC3 and RFP‐p62 expressed in a baculovirus expression system. Recombinant proteins expressed in the three different groups of primary human lung fibroblasts showed similar levels of autophagosomes at steady state. After 24 h of CQ treatment, young fibroblasts displayed significantly higher number of autophagosomes compared to old fibroblasts (Fig. 1C,D). Fibroblasts from patients with IPF showed significantly less LC3‐II flux compared to those of normal controls of the same age as shown for levels of LC3 puncta per cell (Fig. 1C and D). p62 flux measured by this method was significantly lower in old and IPF fibroblasts compared with young fibroblasts although no differences were detected between them. In Fig. S1, we show that fibroblasts from IPF seem to have an incomplete colocalization of p62 with LC3, suggesting that p62 is accumulating out of the autophagosome which might explain the lack of difference in total p62 among old and IPF fibroblasts. However, no quantification was performed.

In addition, expression profile of autophagy‐related genes through Profiler PCR Array Human Autophagy revealed that lung fibroblasts from ‘old’ normal donors have upregulated genes with an inhibitory effect on the autophagy pathway compared with young fibroblasts such as AKT, BCL2L1 (Morselli *et al*., 2009) (Table 1; the complete list of genes are shown in Table S2). Therefore, in human fibroblasts, aging is related to defective autophagy. The inhibitory effect of aging in autophagy was confirmed in old mice (24 months) lung fibroblasts compared to younger (2 months) counterparts (Fig. S2).

**Table 1:** Changes in the expression of autophagy‐related genes with aging^a^

Gene symbol	Fold regulation	p value
AKT1	4.2	0.045
BCL2L1	2.6	0.023
ATG4B	2.5	0.025
HSP90AA1	2.4	0.019
ATG12	2.3	0.020
CASP3	−2.1	0.026

a Complete list in Table S2.

### Aging decreases starvation‐induced autophagy through mTOR activity in fibroblasts from patients with IPF

**Figure 2:**
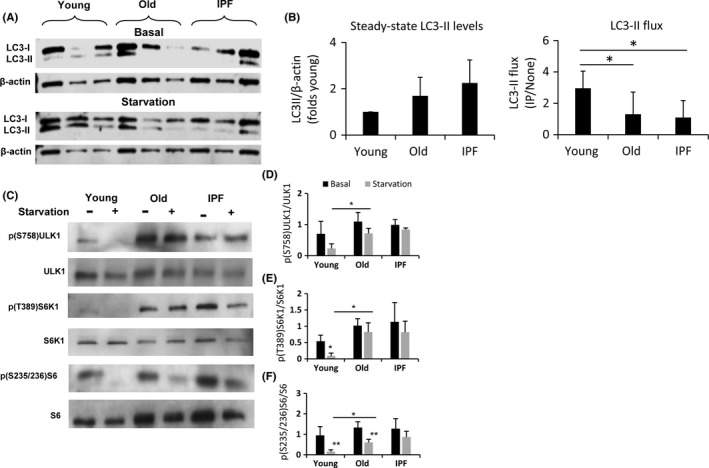
Aging prevents autophagy induction mediated by mTOR pathway. Autophagy and mTOR pathway activity were examined in human fibroblasts derived from young, old, and IPF lungs. (A): Representative Western blot of LC3 and β‐actin as loading control, under basal conditions and after 1 h of starvation with inhibitors of proteases (IP). (B) Densitometric analysis showing steady‐state levels and LC3‐II flux. Each bar represents the mean ± SD of 6 different lines of lung fibroblasts. *P < 0.05 two‐tailed Student's *t*‐test. The activity of mTOR complex 1 was examined by the phosphorylation of ULK1, S6K1, and S6. (C) Immunoblots of p(S758)ULK1, ULK1, p(T389)S6K1, S6K1, p(S235/236)S6, S6 at baseline and after 1 h of starvation. (D, E, F) Densitometric analysis of the phosphorylation ratio of ULK1, S6K1, and S6 shown in C. Each bar represents the mean ± SD of 3 different cell lines for each group in D and E and 6 different cell lines for each group in F. *P < 0.05, **P < 0.01 two‐tailed Student's *t*‐test.

Starvation is the most potent inducer of autophagy (Galluzzi *et al*., 2014), and accordingly, we used starvation to analyze LC3 turnover. Initially, we examined starvation at 0, 30, and 60 min with and without inhibitors of proteases. We choose for our experiments 1‐h starvation. Figure**** 2 **A** illustrates a representative immunoblot of basal and starvation conditions with inhibitors. The basal and starvation levels without inhibitors (data not shown) were considered for the graphical representation (Fig. 2B). Under these conditions, LC3‐II flux was significantly higher in young fibroblasts, compared with old and IPF fibroblasts.

Autophagy induction follows an inverse correlation with the activity of mTOR complex 1. mTOR directly prevents autophagy activation through phosphorylation of Ser 758 in ULK1 (Kim *et al*., 2011). This phosphorylation was evaluated to explore the mechanism by which fibroblasts derived from old individuals and IPF patients show decreased induction of autophagy. At baseline, although phosphorylation is present in young, old, and IPF fibroblasts, after 1 h of starvation, this phosphorylation was inhibited in young fibroblasts, while it was maintained in old and IPF fibroblasts (Fig. 2C–D). These findings suggest that in these fibroblasts, mTOR activation persists as also demonstrated by phosphorylation of (T389) S6K1 (Fig. 2C–E). The deficiency in mTOR inhibition might be responsible for the decrease in autophagy flux observed in old and IPF fibroblasts, and corresponds to a failure of adaptation to stress, as it has been observed in other diseases associated with aging (Johnson *et al*., 2013). The phosphorylation of (S235/236) S6 was reduced in young but also in the group of old fibroblasts, suggesting a possible delay in the inactivation of mTOR pathway. No changes were observed in IPF fibroblasts (Fig. 2C–F).

### Fibroblasts from patients with IPF show a persistent activation of mTOR, which contributes to apoptosis resistance

**Figure 3:**
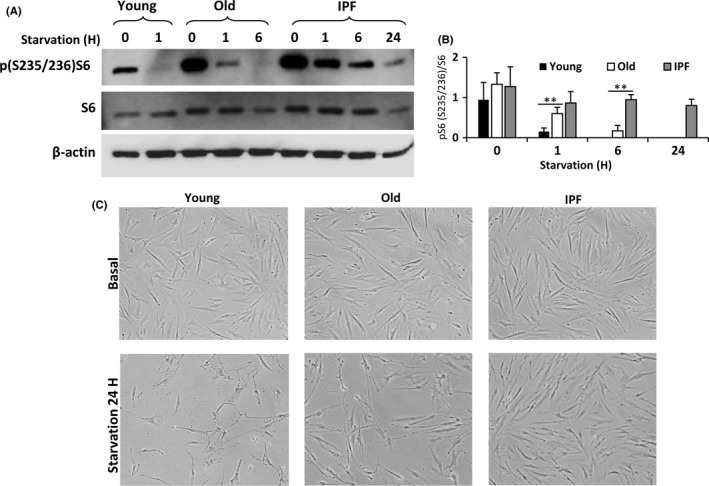
IPF fibroblasts exhibit a persistent activation of mTOR. mTOR pathway activity was examined in normal lung fibroblasts sorted by age and IPF fibroblasts after starvation. (A) Representative Western blot of p(S235/236)S6, S6, and β‐actin as a loading control in fibroblasts exposed for different hours of HBSS media to induce starvation. (B) Densitometric analysis of the immunoblot showing the phosphorylation of S6. Each bar represents the mean ± SD of 6 different cell lines for each group in the time 0 and 1 h; and 3 different cell lines for each group in the time 6 and 24 h. **P < 0.01 two‐tailed Student's *t*‐test. (C) Representative micrographs of cells from young, old, and IPF fibroblasts at baseline and after 24 of starvation.

**Figure 4:**
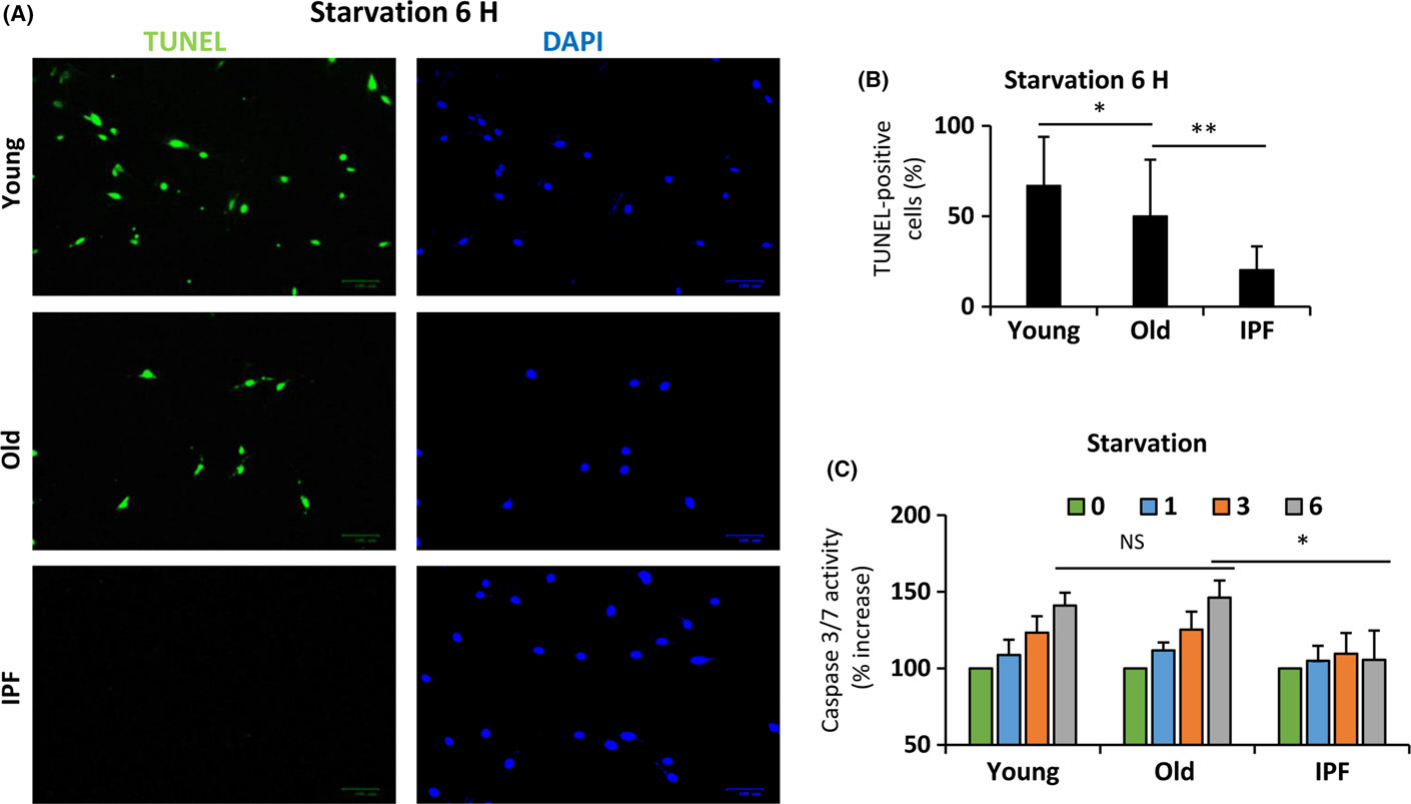
IPF fibroblasts show diminished induction of apoptosis after starvation. Normal human lung fibroblasts sorted by age and IPF fibroblasts were exposed to HBSS media to evaluate the induction of apoptosis. (A) Detection of apoptotic cells using TUNEL stain and DAPI for nuclear staining in fibroblasts exposed to 6 h of starvation. (B) Percentage of TUNEL‐positive cells adjusted by the total number of cells quantified by DAPI. Each bar represents the mean ± SD of 3 different cell lines in each group (n > 150 cells per group). *P < 0.05, **P < 0.01 two‐tailed Student's *t*‐test. (C) Activity of caspases 3/7 under basal conditions (0) and after 1, 3, and 6 h of starvation. Data are expressed as mean ± SD of 3 different cell lines from two independent experiments. *P < 0.05, two‐tailed Student's *t*‐test.

Next, we investigated the effect of increased exposure time of starvation on mTOR pathway in old and IPF fibroblasts. As previously shown, at 1 h of starvation mTOR activity determined by phosphorylation of S6 was abolished in young fibroblasts. At 6 h under these conditions, complete abrogation of mTOR activity was observed in old normal fibroblasts, but not in IPF‐derived cells, which exhibited a persistent activation even at 24 h of starvation (Fig.**** 3A–B). mTOR is a key regulator in the adaptation to nutrients stress and persistent mTOR activity under nutrient deprivation can mediate changes in cell survival. Microscopic observation of fibroblasts suggested that prolonged exposure to starvation induces cell death in young and old normal fibroblasts, but not in IPF fibroblasts (Fig. 3C). The decreased apoptotic response in IPF fibroblasts was confirmed by TUNEL assay and activity of caspases 3/7 after starvation. As illustrated in Fig. 4A,B, young and old fibroblasts showed abundant apoptotic positive signal, whereas the signal was almost undetectable in IPF fibroblasts. Paralleling these results, the activity of caspase increased in a time‐dependent manner in young and old but not in IPF fibroblasts (Fig. 4C).

### mTOR inhibitors modify starvation‐induced autophagy in old and IPF fibroblasts

mTOR complexes 1 and 2 regulate the starvation‐stress response. mTORC1 activation increases protein translation and other anabolic processes with direct inhibition of autophagy (Galluzzi *et al*., 2014). mTORC2 regulates Akt, SGK1, and PKCα (Laplante & Sabatini, 2012). Evidence indicates that there is a feedback between both complexes. Rapamycin is an allosteric inhibitor of mTORC1 and PP242 is a new drug with potent and selective inhibitory effect on ATP domain of mTOR that suppresses its activity in both complexes (Lamming *et al*., 2013).

The effect of both inhibitors was analyzed through the phosphorylation of S6K1 and S6 in IPF fibroblasts. When treated with rapamycin for 24 h, IPF fibroblasts displayed a modest reduction of mTOR activity, while PP242 showed a marked effect as demonstrated by the strong decrease in phosphorylation of S6K1 and S6 (Fig. S3).

**Figure 5:**
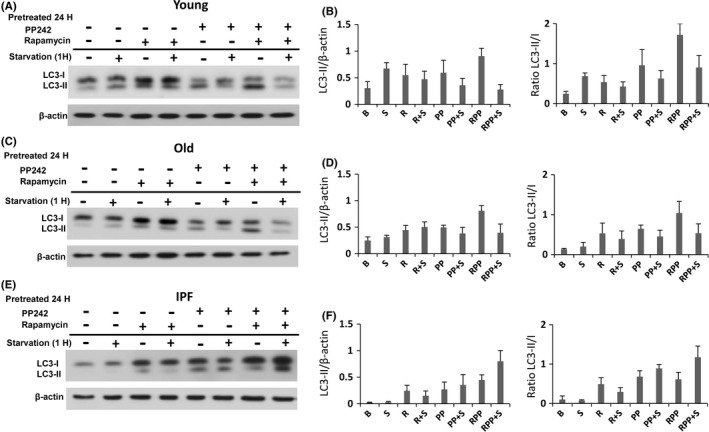
Effect of rapamycin (20 nM) and/or PP242 (1uM) for 24 h on autophagy activity in the three groups of lung fibroblasts (young, old, and IPF). The effect on autophagy with and without starvation was evaluated by Western blot for LC3 using β‐actin as a loading control in young (A), old (C), and IPF‐derived fibroblast (E). (B, D, F) Densitometric analysis of LC3II Western blots presented in A, C, and E. Each bar represents the mean ± SD of 2 different cell lines for each group.

Treatment with the pharmacological inhibitors on the autophagy activity corroborated that under starvation, fibroblasts from young lungs exhibited an increase in the LC3‐II/β‐actin and in LC3‐II/LC3‐I ratios. Similar results were observed with the pretreatment of rapamycin, PP242, and mainly with the combination of both (Fig. 5A,B). The noticeable decrease in LC3‐II/β‐actin and LC3‐II/LC3‐I ratios with the combination of inhibitors under starvation may be explained by the reactivation of mTORC activity as shown in Fig. S4. Old fibroblasts, as previously demonstrated, do not increase autophagy under starvation; however, with the pretreatment of the inhibitors, they performed a similar response as young fibroblasts (Fig. 5C–D). In the case of IPF fibroblasts, which are unable to increase autophagy under starvation, pretreatment with the inhibitors mainly with the combination of both and starvation resulted in an increase in LC3‐II/β‐actin and in LC3‐II/LC3‐I ratios (Fig.**** 5E–F). By contrast to young and old fibroblasts, mTORC activity in IPF fibroblasts was not reactivated with the combination of rapamycin plus PP242 under starvation (Fig. S4).

### mTOR pathway inhibitors modify the apoptotic response in IPF fibroblasts

**Figure 6:**
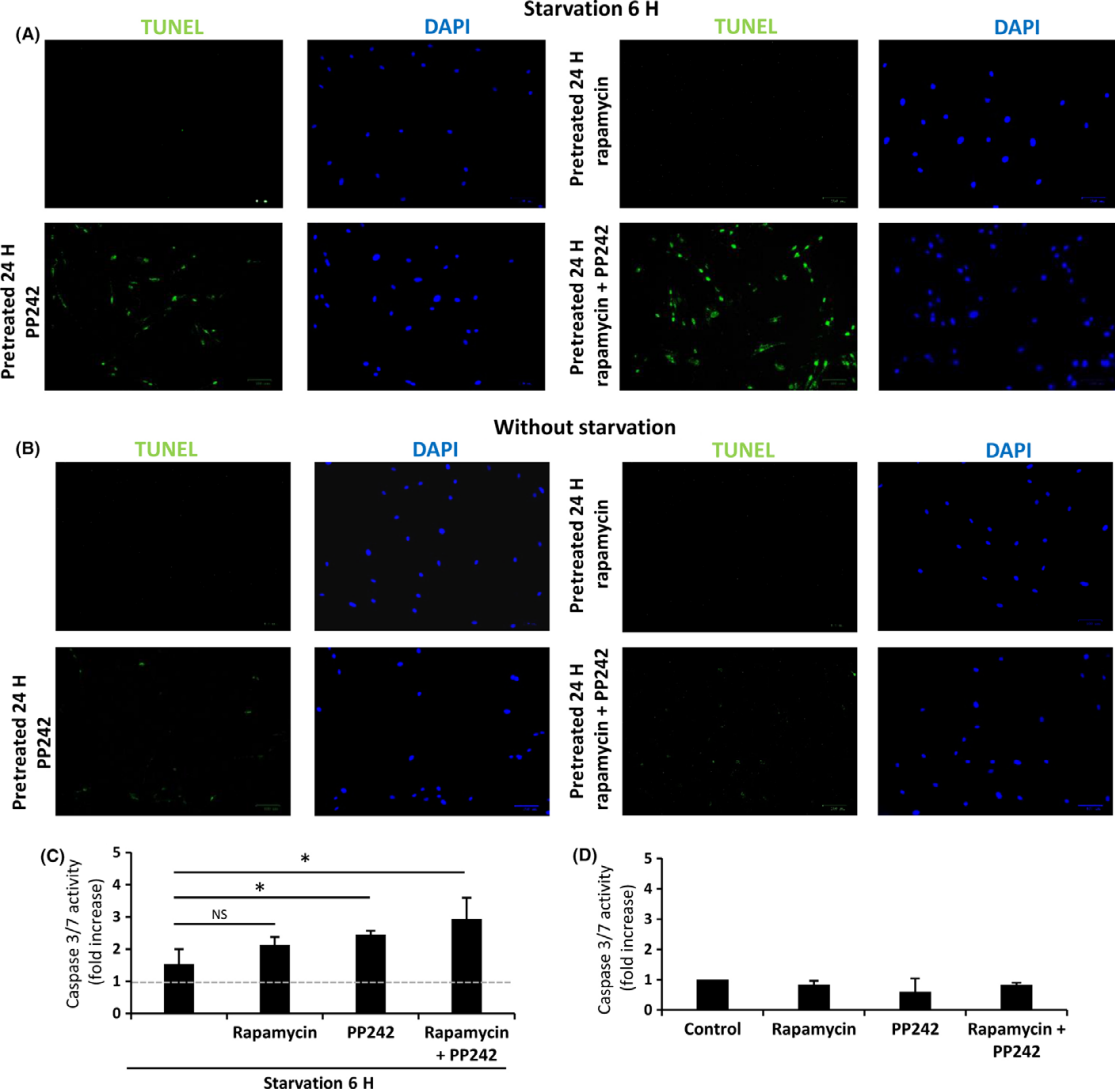
mTOR pathway inhibitors increase the apoptotic response in IPF fibroblasts. Detection of apoptotic cells using TUNEL stain and DAPI for nuclear staining under 6 h of starvation (A) and basal conditions (B). (C, D) Activity of caspases 3/7 with and without starvation in IPF fibroblasts. Data are expressed as mean ± SD of 2 different lines of IPF fibroblasts. *P < 0.05, two‐tailed Student's *t*‐test.

Next, we evaluated whether the decreased apoptotic response observed in IPF fibroblasts as shown in Fig. 4 was affected by mTOR inhibitors. After 6‐h starvation, the inhibition of mTOR with PP242 alone or in combination with rapamycin induced an increase in the number of cells showing positive apoptotic signal by TUNEL (Fig. 6A), while on basal nonstarving conditions, no differences were observed (Fig. 6B). Likewise, a significant increase in caspase 3/7 activity in IPF fibroblasts was detected after 6 h of starvation, mainly with the combination of PP242 and rapamycin (Fig. 6C), whereas no differences were noticed under basal conditions (Fig. 6D). Rapamycin alone induced a marginal but nonsignificant increase in these caspases.

## Discussion

Aging is not a disease itself, but the biological processes that change with age have a crucial role in numerous chronic degenerative diseases (Kennedy *et al*., 2014). One common feature in age‐related diseases such as cancer, cardiovascular disorders, and neurodegenerative diseases is autophagy dysfunction (Rubinsztein *et al*., 2012; He *et al*., 2013). Moreover, there is growing evidence supporting that autophagy deficiency recapitulates aging phenotype (Cuervo *et al*., 2005; Rubinsztein *et al*., 2011).

In lung diseases, aging increases morbidity and mortality of chronic obstructive pulmonary disease, pneumonias, and IPF, but the involved mechanisms are not fully elucidated (Ryter & Choi, 2010; Selman & Pardo, 2014; Thannickal *et al*., 2015). Along this line, IPF is the prototype of age‐associated disease since usually occurs in individuals older than 50 years old, and increases remarkably with aging (Gribbin *et al*., 2006; Raghu *et al*., 2011). Actually, aging is the higher demographic risk factor for IPF. Several mechanisms seem to be implicated, but all of them have been revealed in alveolar epithelial cells including abnormal shortening of telomeres, genomic instability, and senescence (Selman & Pardo, 2014). More recently, we have shown that mitochondrial dysfunction associated with PINK1 deficiency in alveolar epithelial cells with age and ER stress promotes fibrosis (Bueno *et al*., 2015). However, the effect of aging in IPF fibroblasts remains unclear.

In this context, the present study was designed to examine the putative role of aging in autophagy on normal lung fibroblasts as well as on IPF fibroblasts. We found that aging inhibits autophagy of human and mouse lung normal fibroblasts. However, we observed few differences between old and IPF fibroblasts exemplified by the detection of LC3 puncta per cell. Compared with old normal lung fibroblasts, IPF fibroblasts showed less amount of autophagosomes with a possible improper p62 localization. Under stress conditions, mTOR pathway in old fibroblast stays active for a longer period which was even more accentuated in IPF fibroblasts. This persistent activity in IPF cells match with an increased resistance to apoptosis.

Autophagy flux is critical to maintain cell homeostasis. Our results show that aged normal lung and IPF fibroblasts have a dysfunction in autophagy activity. On basal conditions, some cases of old and IPF fibroblasts have an interrupted autophagy flux. When accumulation is induced, there is less activity of autophagy in old and IPF fibroblasts, analyzed by two different methods and confirmed in lung fibroblasts from aging mice. In addition, genes that participate in the regulation of autophagy are dysregulated in old fibroblasts. For example, AKT, which is involved in activation of mTOR, and BCL2L1 that sequesters beclin 1, are overexpressed in old fibroblasts with a negative regulation of autophagy (Shaw & Cantley, 2006; Maiuri *et al*., 2007). The upregulation of ATG4b in old fibroblasts, which is involved in lipidation of LC3, may represent a ‘compensatory’ mechanism that prevents fibrosis. Actually, a recent study performed in our laboratory demonstrates that ATG4b has a protective role in experimental fibrosis (Cabrera *et al*., 2015).

Hormesis is a beneficial approach because it engages in the protective mechanisms to overcome stress (Matus *et al*., 2012). For instance, caloric restriction has been reported that increases longevity mediated by autophagy (Kroemer, 2015). Based on these studies, we induced stress by a short period of starvation. We found that young fibroblasts show the expected increase in autophagy activity and inhibition of mTOR activity, while old and IPF fibroblasts showed persistent activation of mTOR and amelioration of autophagy. Interestingly, the phosphorylation of (S235/236) S6 was reduced in old fibroblasts, from the first hour of starvation and abolished at 6 h, suggesting a possible delay in the inactivation of mTOR pathway. In sharp contrast, IPF‐derived cells exhibited a persistent activation of mTOR even at 24 h of starvation which may contribute to apoptosis resistance.

Pretreatment with rapamycin, PP242, or in combination resulted in an increase in autophagy in young, old, and IPF fibroblasts. While rapamycin had a modest inhibition of mTOR pathway, PP242 or the combination of both inhibitors showed a stronger effect. PP242 is a novel and specific ATP competitive inhibitor of mTOR kinase that is a dual inhibitor of TORC1 and TORC2 and inhibits more efficiently TORC1 than rapamycin (Feldman *et al*., 2009). Further studies are necessary to investigate the specific mTOR complex involved.

Autophagy and apoptosis are processes interconnected in stress responses. Normally under stress, these processes have a synchronous response with an early autophagic stage and a late apoptotic stage (Marino *et al*., 2014). Following this approach, apoptosis was determined under prolonged starvation. Remarkably, IPF fibroblasts displayed persistent activation of mTOR pathway that confers them resistance to apoptosis.

Altogether, our results suggest that in IPF fibroblasts, both processes decreased autophagy activity and increased resistance to apoptosis may be linked. Thus, when mTOR activity was inhibited, autophagy was increased and also apoptosis, and moreover, stronger increase in autophagy was accompanied by stronger induction of apoptosis. However, as regulation of autophagy and death are both downstream of mTOR, we cannot rule out that these two processes might not be mechanistically linked.

It is well known that autophagy and apoptosis cross‐regulate each other, usually in an inhibitory manner. Thus, autophagy reduces the susceptibility of cells to undergo apoptosis, while apoptosis suppresses autophagy (Mattiolo *et al*., 2015). However, there are also several examples in which the induction of autophagy facilitates the activation of apoptosis, and our findings suggest that this is what occurs in IPF fibroblasts.

We propose that this failure in the response to starvation could play a key role in the pathophysiology of IPF which is characterized by the persistence of the fibroblasts organized in the fibroblastic foci. These foci represent sites of active fibrogenesis and are critical for the fibrotic progression. Actually, the decrease in intracellular degradation by autophagy and the interruption of apoptosis both mediated by aberrant activation of mTOR pathway are consistent with the IPF fibroblast phenotype. Supporting the notion that decreased autophagy activity contributes to fibrosis, we have found that knockout mice for MMP‐19, when exposed to bleomycin instillation, develop a stronger lung fibrotic response with the formation of fibroblasts foci. These fibroblasts exhibited a decrease in the expression of Atg4c, other member involved in autophagy stress response that has been associated with aging (Fernandez & Lopez‐Otin, 2015; Jara *et al*., 2015).

Altogether, our results indicate that age is a determinant factor in the autophagy response of lung fibroblasts. Our data support that age‐related deficiency in adaptive responses to stress, together with an increased resistance to apoptosis of IPF lung fibroblasts, mediated by mTOR activity, enhances the vulnerability to lung fibrosis.

## Experimental procedures

### Human lung fibroblasts culture

The study was approved by the University of Pittsburgh Institutional Review Board and. Lung tissues from normal controls were provided by the PACCM Bio‐Bank and Instituto Nacional de Enfermedades Respiratorias (INER) under protocols approved by the University of Pittsburgh Institutional Review Board and the ethics committees of INER with a written informed consent obtained from all participating individuals. Lung fibroblasts were insolated by enzymatic dispersion with trypsin (Sigma‐Aldrich). Cells were grown with Ham's F‐12 (Gibco) add 10% FBS (Gibco) at 37 °C in an atmosphere of 95% air and 5% CO_2_ until reaching early confluence from the passage 4 to 8.

### Reagents

To induce starvation, cells were incubated in Hank's balanced salt solution (HBSS) (Gibco). Protease inhibitors (Sigma‐Aldrich) 1:500 or chloroquine (Sigma‐Aldrich) 20uM were used to induce the accumulation of autophagosomes. Rapamycin 20 nM (Sigma‐Aldrich) and/or PP242 1uM (Sigma‐Aldrich) were used to inhibit mTOR complexes 1 and 2.

### Western blot

Proteins were extracted using RIPA (Sigma), and 20ug of total protein was used for electrophoresis in a polyacrylamide gel. Proteins were transferred to PVDF membrane and blocked for 1 h. Primary antibodies for LC3 (Sigma), P62 (Abcam), p(S758)ULK1, ULK1, p(T389)S6K1, S6K1, p(S235/236)S6, S6 (cell signaling), and beta‐actin as loading control (Sigma) were incubated overnight. Finally, secondary antibodies (Licor) and Odyssey scanner (Licor) were used. Quantification was performed using ImageJ software (NIH).

### Quantitative PCR (qPCR)

About 5ug of total RNA was isolated with Trizol (Sigma), according to the manufacturer's instructions. cDNA synthesis and genomic DNA elimination were done with RT² First Strand Kit (Qiagen). Gene expression analysis was performed using RT² Profiler PCR Array Human Autophagy (Qiagen) with 84 autophagy‐related genes simultaneously with CFX96 Real Time PCR System (Bio‐Rad). Data analysis was based on the 2^−ΔCT^ method with normalization of the housekeeping genes.

### Analysis of autophagy activity by fluorescence microscopy

Cells were incubated overnight with a baculovirus for LC3B‐GFP and p62‐RFP (Premo^™^ Autophagy Sensors, Invitrogen). To induce the accumulation of autophagosomes, CQ (20uM) was used for 24 h. The microphotographs were taken of live cells with an inverted epifluorescence microscope (eclipse, NIKON) with an objective PlanFLUO 60X. Quantification was performed using NIS software (Nikon), and LC3 intense dots were quantified for each cell and plotted as the average of 5 photos of two independent experiments per cell line (*n* > 30 cells per group).

### Apoptosis

The cells were incubated with HBSS (Gibco) to induce starvation. Number of apoptotic cells was determined with TUNEL stain. Cells were fixed with 1% paraformaldehyde and ApopTag kit (Millipore) was used according to manufacturer's instructions. Nuclear stain was through VECTASHIELD mounting media with DAPI (Vector). The microphotographs were taken using ZOE^™^ Fluorescent Cell Imager (Bio‐Rad). Positive cells for TUNEL and DAPI were quantified and plotted as the average of 5 photographs of two independent experiments per cell line (*n* > 150 cells per group).

### Caspase 3/7 activity assay

The activity of caspases 3 and 7 was evaluated using the caspase‐glo 3/7 assay (Promega) according to the manufacturer's instructions. Briefly, the luminescent substrate is released by the cleavage of caspases and the luminescent signal was measured in a GloMax luminometer (Promega).

### Statistical analysis

Two‐tailed Student's *t‐*test was used to determine statistical significance.

## Funding

The present study was supported by PAPIIT IN214612‐3, CONACYT 251636, NIH RHL131789A, and NIH R01 HL123766‐01A1 and by Vascular Medicine Institute, University of Pittsburgh, the Institute for Transfusion Medicine, and the Hemophilia Center of Western Pennsylvania.

## Author contributions

All authors participated in the revision of the manuscript. YR, ALM, and AP performed study conception and design; YR, RR, DA, and JS carried out acquisition of data; YR, MB, EAG, MR, MS, ALM, and AP carried out analysis and interpretation of data; YR, MR, MS, ALM, and AP drafted the manuscript.

## Conflict of interest

None declared.

## Supporting information


**Fig. S1** IPF fibroblasts show an incomplete LC3 and p62 colocalization after chloroquine treatment. Fluorescence micrographs of baculovirus infection of LC3‐GFP and p62‐RFP in the three groups of fibroblasts after chloroquine treatment (20uM). Hoechst was added to nuclei stain.

**Fig. S2** Aging decreases autophagy markers in lung fibroblasts from old (24 months) compared with young (2 months) mice. Western blots of LC3, p62, Atg5, Beclin‐1 after 1 h of starvation or chloroquine treatment (20uM) (A). (B): Same treatment for 24 h. β‐actin was used as a loading control. Densitometric analysis of (A) and (B) blots are showed in C and D, respectively. Each bar represents the mean ± SD of two different cell lines.

**Fig. S3** Rapamycin and/or PP242 reduce the activity of mTOR in IPF fibroblasts. (A) Fibroblasts from IPF patients were stimulated with rapamycin (20 nM) and/or PP242 (1uM) for 24 h and the activity of mTOR complex 1 was examined by Western blot through the phosphorylation of (T389) S6K1 and (S235/236) S6. (B, C): Densitometric analysis. Each bar represents the mean ± SD of 3 different cell lines for each group. * *P* < 0.05 two‐tailed Student's *t*‐test.

**Fig. S4** Effect of rapamycin plus PP242 and starvation on mTOR activity in young and IPF fibroblasts. Fibroblasts from young lungs (A) and IPF patients (B) were stimulated with rapamycin (20 nM) and PP242 (1uM) for 24 h with and without starvation and the activity of mTOR complex 1 was examined by Western blot through the phosphorylation of (S235/236) S6.

**Table S1** Age and sex of enrolled subjects and patients with IPF.

**Table S2** Changes in the expression of autophagy‐related genes with aging (complete list).

**Data S1** Supplementary experimental procedures.
