# Supplementary figures and images for: mTORC1 activation decreases autophagy in aging and idiopathic pulmonary fibrosis and contributes to apoptosis resistance in IPF fibroblasts
Source: Aging Cell. 2016 Aug 26;15(6):1103–12. doi: 10.1111/acel.12514 (PMC6398527; doi:10.1111/acel.12514)

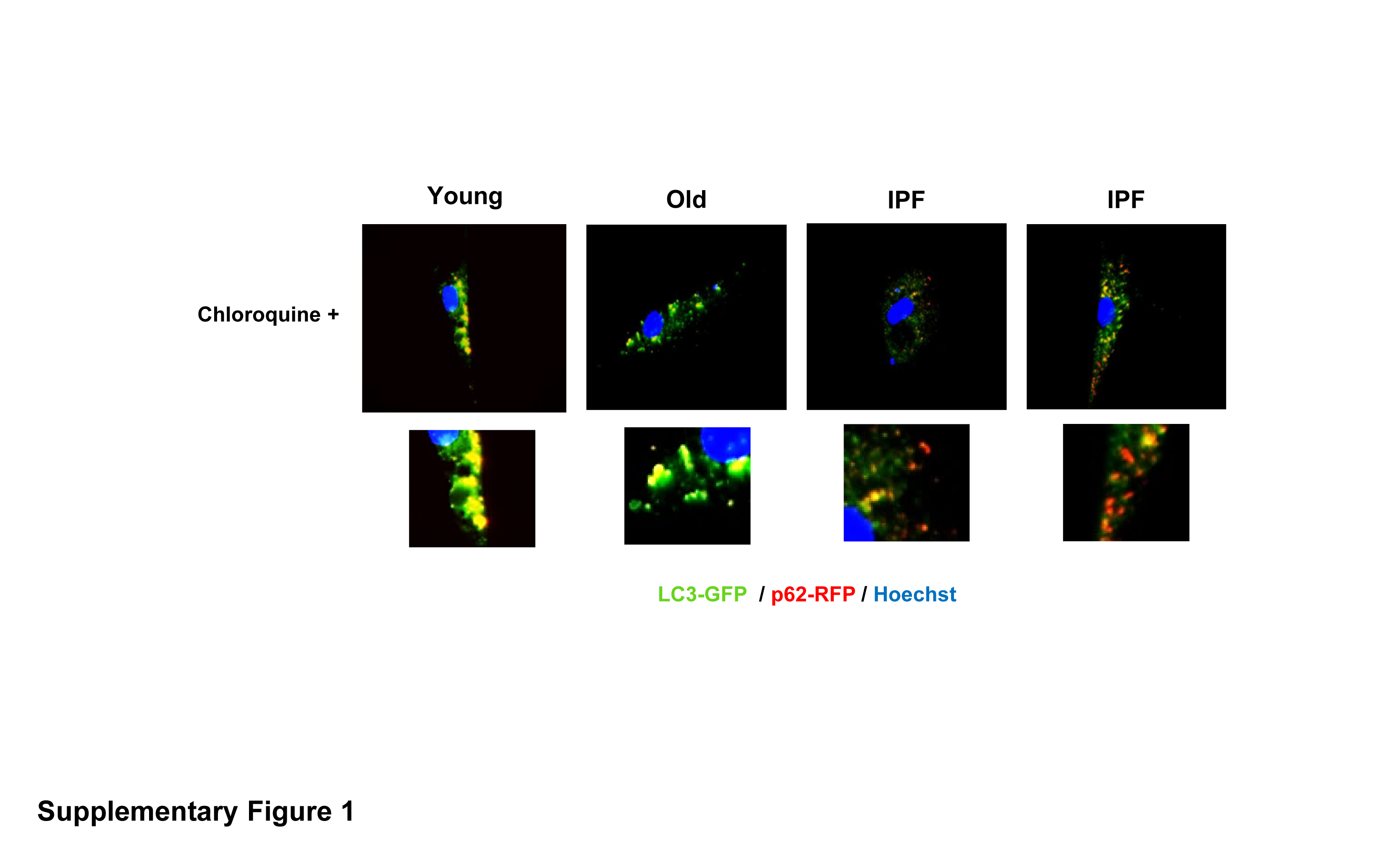

Supplement: Supplementary file 1 — Fig. S1 IPF fibroblasts show an incomplete LC3 and p62 colocalization after chloroquine treatment. Fluorescence micrographs of baculovirus infection of LC3‐GFP and p62‐RFP in the three groups of fibroblasts after chloroquine treatment (20uM). Hoechst was added to nuclei stain. [file ACEL-15-1103-s001.jpg]

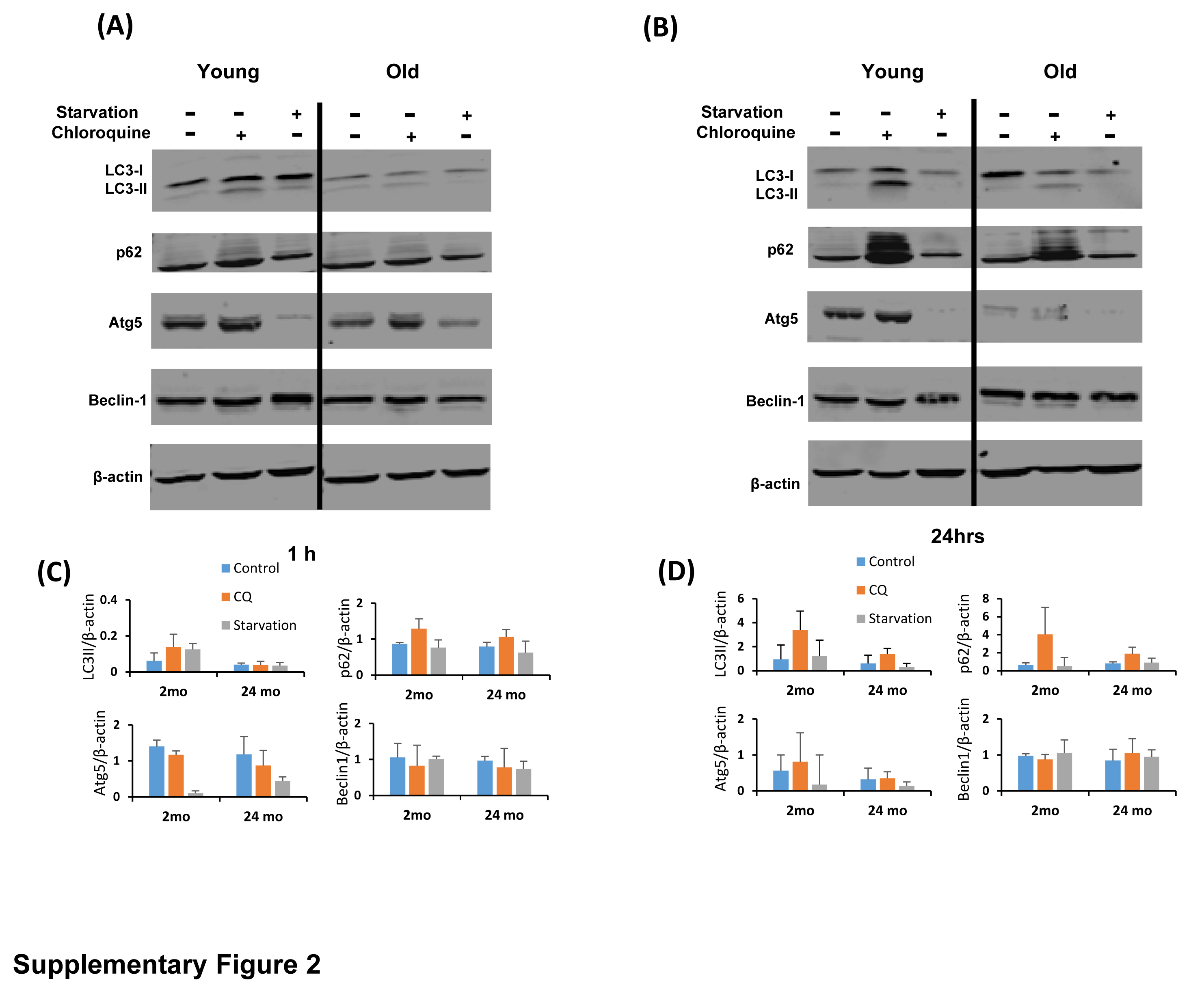

Supplement: Supplementary file 2 — Fig. S2 Aging decreases autophagy markers in lung fibroblasts from old (24 months) compared with young (2 months) mice. Western blots of LC3, p62, Atg5, Beclin‐1 after 1 h of starvation or chloroquine treatment (20uM) (A). (B): Same treatment for 24 h. β‐actin was used as a loading control. Densitometric analysis of (A) and (B) blots are showed in C and D, respectively. Each bar represents the mean ± SD of two different cell lines. [file ACEL-15-1103-s002.jpg]

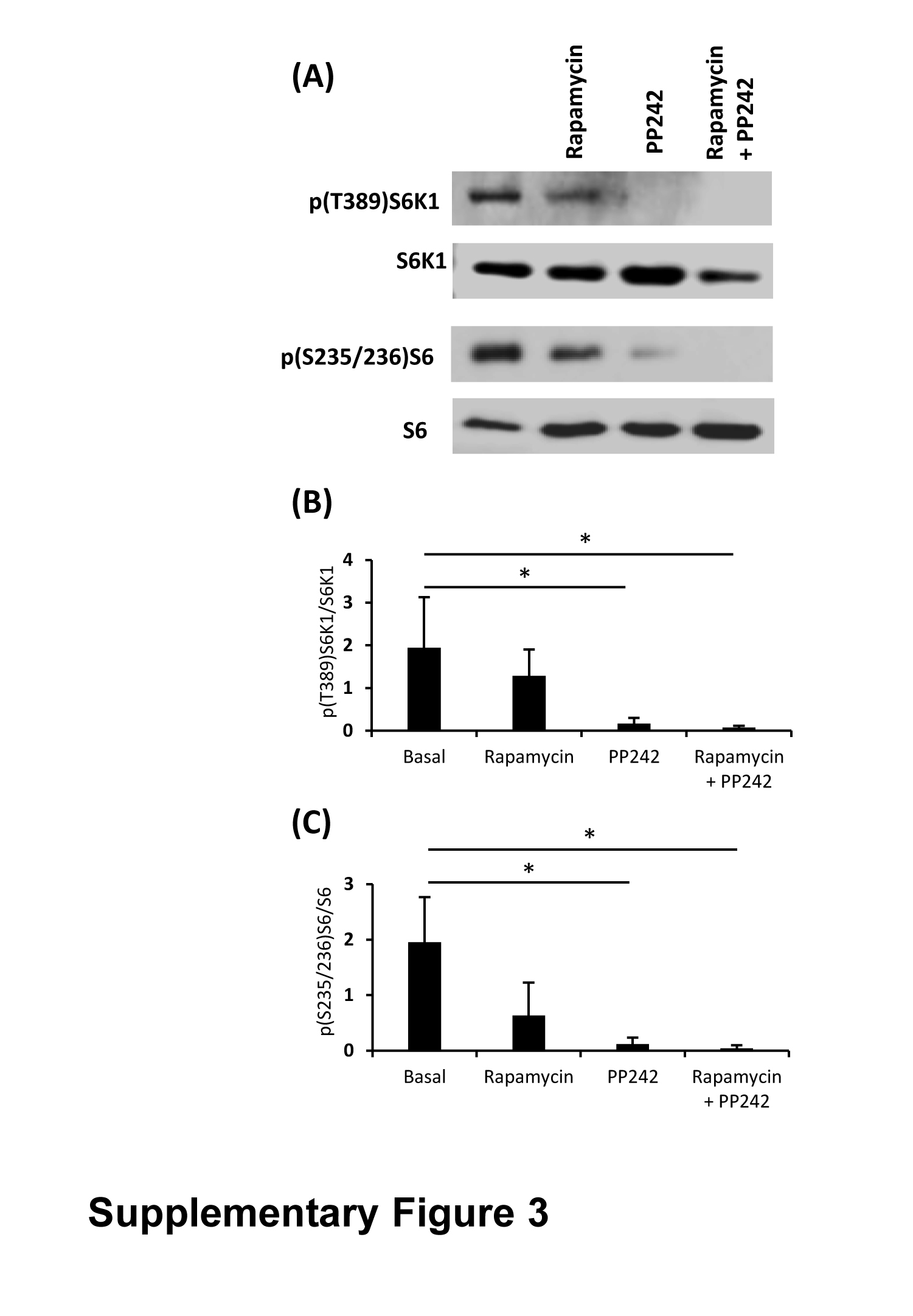

Supplement: Supplementary file 3 — Fig. S3 Rapamycin and/or PP242 reduce the activity of mTOR in IPF fibroblasts. (A) Fibroblasts from IPF patients were stimulated with rapamycin (20 nM) and/or PP242 (1uM) for 24 h and the activity of mTOR complex 1 was examined by Western blot through the phosphorylation of (T389) S6K1 and (S235/236) S6. (B, C): Densitometric analysis. Each bar represents the mean ± SD of 3 different cell lines for each group. * P < 0.05 two‐tailed Student's t‐test. [file ACEL-15-1103-s003.jpg]

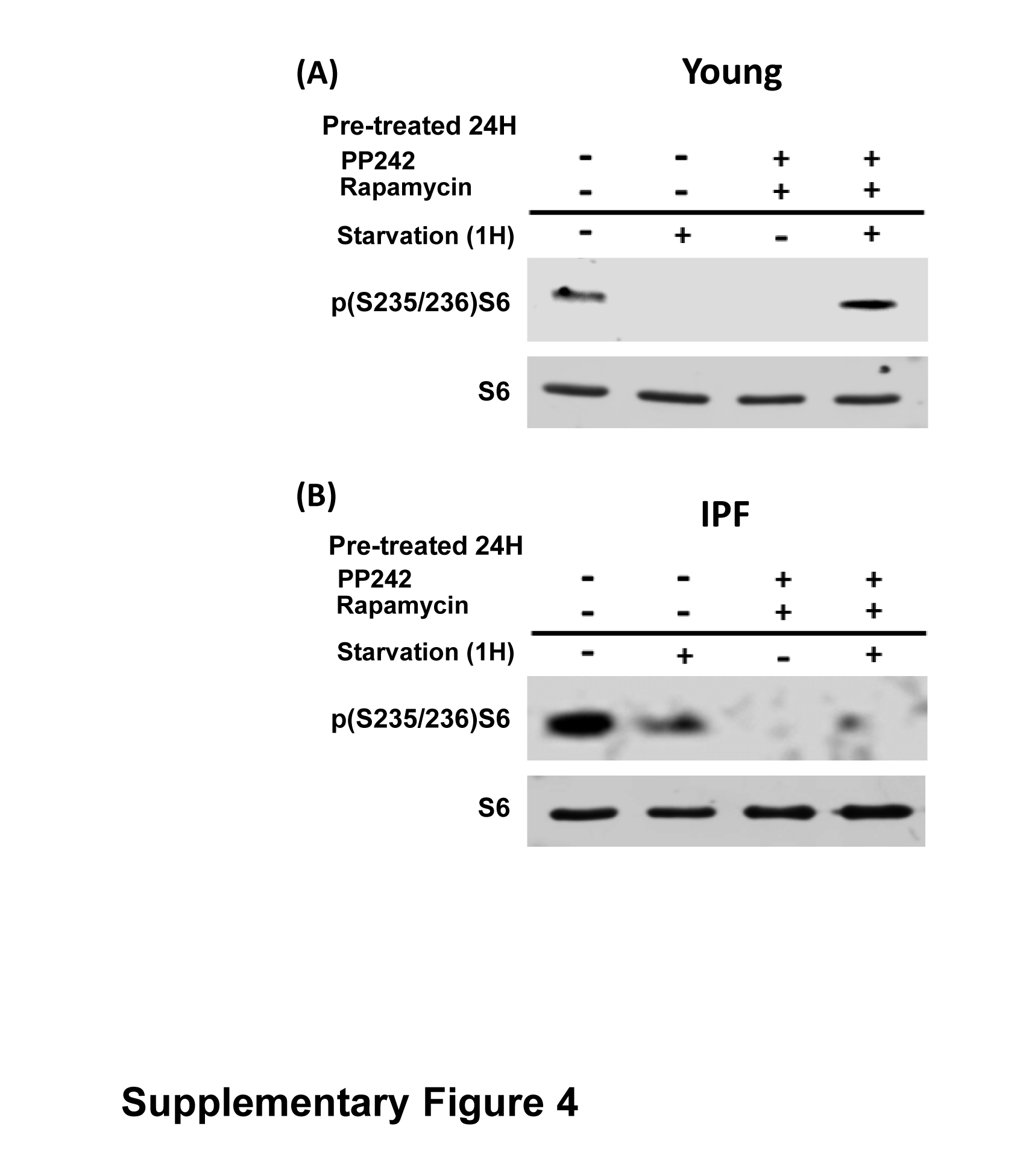

Supplement: Supplementary file 4 — Fig. S4 Effect of rapamycin plus PP242 and starvation on mTOR activity in young and IPF fibroblasts. Fibroblasts from young lungs (A) and IPF patients (B) were stimulated with rapamycin (20 nM) and PP242 (1uM) for 24 h with and without starvation and the activity of mTOR complex 1 was examined by Western blot through the phosphorylation of (S235/236) S6. [file ACEL-15-1103-s004.jpg]
