# Supplementary material for: mTORC1 activation decreases autophagy in aging and idiopathic pulmonary fibrosis and contributes to apoptosis resistance in IPF fibroblasts
Source: Aging Cell. 2016 Aug 26;15(6):1103–12. doi: 10.1111/acel.12514 (PMC6398527; doi:10.1111/acel.12514)
Supplement: Supplementary file 5 — Table S1 Age and sex of enrolled subjects and patients with IPF. [file ACEL-15-1103-s005.pdf]

Table S1. Age and sex of enrolled subjects and patients with IPF

Group 1	Age	Sex	Group 2	Age	Sex	Group 3	Age	Sex
Young 1	15	F	Old 1	54	M	IPF 1	60	M
Young 2	16	M	Old 2	64	M	IPF 2	64	M
Young 3	33	F	Old 3	68	M	IPF 3	67	M
Young 4	33	F	Old 4	70	M	IPF 4	68	M
Young 5	33	M	Old 5	71	M	IPF 5	68	M
Young 6	42	M	Old 6	72	M	IPF 6	70	M
**Mean**	**29**		**Mean**	**67**		**Mean**	**66**	
**SD**	**11**		**SD**	**7**		**SD**	**4**	
