# Supplementary material for: mTORC1 activation decreases autophagy in aging and idiopathic pulmonary fibrosis and contributes to apoptosis resistance in IPF fibroblasts
Source: Aging Cell. 2016 Aug 26;15(6):1103–12. doi: 10.1111/acel.12514 (PMC6398527; doi:10.1111/acel.12514)
Supplement: Supplementary file 6 — Table S2 Changes in the expression of autophagy‐related genes with aging (complete list). [file ACEL-15-1103-s006.pdf]

Table S2. Changes in the expression of autophagy-related genes with aging (complete list).

Gene Symbol	Fold regulation	p value
AKT1	4.2	**0.045**
ATG16L1	3.2	0.077
TNF	3.2	0.106
BCL2L1	2.6	**0.023**
ATG4B	2.5	**0.025**
FAM176A	2.5	0.064
HSP90AA1	2.4	0.019
ATG12	2.3	**0.02**
SQSTM1	2.1	0.103
EIF4G1	2	0.152
HSPA8	2	0.182
BCL2	2	0.051
TGM2	1.9	0.353
SNCA	1.8	0.461
IRGM	1.8	0.252
ULK1	1.7	0.234
ARSA	1.7	0.258
TMEM74	1.7	0.18
GABARAPL1	1.7	0.26
ATG9B	1.7	0.35
UVRAG	1.7	0.108
GAA	1.6	0.221
BAX	1.6	0.19
TGFB1	1.6	0.199
MAPK14	1.5	0.095
CTSB	1.4	0.684
TP53	1.4	0.337
ATG16L2	1.4	0.369
PIK3CG	1.4	0.285
ATG9A	1.4	0.619
HGS	1.4	0.301
ATG3	1.3	0.392
FAS	1.3	0.385
FADD	1.3	0.387
ATG4C	1.3	0.284
ATG5	1.3	0.301
DRAM1	1.3	0.301
PIK3R4	1.3	0.301
ESR1	1.3	0.586
CLN3	1.3	0.359
TP73	1.3	0.671
IFNA2	1.2	0.651
ATG4A	1.2	0.704
MAP1LC3A	1.2	0.685
RGS19	1.2	0.451
ATG10	1.2	0.407
CTSS	1.2	0.94
ATG4D	1.2	0.513
PRKAA1	1.2	0.482
IFNA4	1.1	0.875
APP	1.1	0.861
BAK1	1.1	0.641
BECN1	1.1	0.745
NFKB1	1.1	0.93
MAPK8	1.1	0.613
PIK3C3	1.1	0.683
RB1	1.1	0.613
MAP1LC3B	1.1	0.978
BAD	1	0.927
CDKN2A	1	0.766
EIF2AK3	1	0.975
RPS6KB1	1	0.927
DRAM2	1	0.927
CASP8	1	0.928
CXCR4	1	0.717
RAB24	1	0.813
HDAC1	1	0.939
HTT	-1.1	0.65
BID	-1.1	0.987
CDKN1B	-1.1	0.528
GABARAP	-1.1	0.927
IFNG	-1.1	0.452
PRKAA2	-1.2	0.516
AMBRA1	-1.2	0.823
PTEN	-1.2	0.279
GABARAPL2	-1.3	0.794
TNFSF10	-1.5	0.377
ATG7	-1.5	0.077
BNIP3	-1.6	0.19
IGF1	-1.6	0.386
INS	-1.7	0.333
ULK2	-2.1	0.183
CASP3	-2.1	**0.026**
DAPK1	-2.2	0.123
