# Supplementary material for: mTORC1 activation decreases autophagy in aging and idiopathic pulmonary fibrosis and contributes to apoptosis resistance in IPF fibroblasts
Source: Aging Cell. 2016 Aug 26;15(6):1103–12. doi: 10.1111/acel.12514 (PMC6398527; doi:10.1111/acel.12514)
Supplement: Supplementary file 7 — Data S1 Supplementary experimental procedures. [file ACEL-15-1103-s007.docx]

**Supporting Information**

**Supplementary experimental procedures**

**Mouse lung fibroblasts culture**

Male mice of C57BL/6 strain of 2 months and 24 months from Jackson Laboratories and National Institute of Aging, respectively, were used to isolate fibroblasts by enzymatic dispersion with trypsin (Sigma-Aldrich) and placed in DMEM (Gibco) with 10% FBS (Gibco). Cells were grown in T-25 flask at 37°C in an atmosphere of 95% air and 5% CO2 until reaching early confluence.

**Western blot**

Protein were extracted using RIPA (Sigma), 20ug of total protein was used for electrophoresis in a polyacrylamide gel 13.5%. Proteins were transferred to PVDF membrane and blocked for 1 hour. Primary antibodies for LC3 (Sigma-Aldrich), P62 (Abcam), ATG5 (Cell Signal), beclin 1 (Cell Signal), beta-actin and beta-tubulin as loading controls (Santa Cruz) were incubated overnight. Finally, secondary antibodies (Licor) and Odyssey scanner (Licor) was used. Quantification was performed using ImageJ software (NIH).
